# Clinical differences between destination therapy and bridge-to-transplant left ventricular assist device implantation: a 17-year single-center experience in Japan

**DOI:** 10.1007/s10047-026-01581-8

**Published:** 2026-08-21

**Authors:** Koichi Inoue, Daisuke Yoshioka, Yusuke Misumi, Ai Kawamura, Shin Yajima, Takuji Kawamura, Shunsuke Saito, Takashi Yamauchi, Shigeru Miyagawa

**Affiliations:** Department of Cardiovascular Surgery, The University of Osaka Graduate School of Medicine, 2-2 Yamadaoka, Suita City, Osaka 565-0871 Japan

**Keywords:** Left ventricular assist device, Destination therapy, End stage heart failure

## Abstract

The use of left ventricular assist device (LVAD) implantation for destination therapy (DT) has been recently increased worldwide. In 2021, DT was also approved in Japan. Here, we report a single-institution experience of LVAD implantation for DT. This retrospective observational study was conducted at Osaka University Medical Hospital in Japan. Forty patients who underwent LVAD implantation for DT were included. The primary endpoint was on-device survival, and the secondary endpoint was the incidence of LVAD-related complications at five years. The on-device survival rates were 82.3%, 73.3%, and 49.8% at 1, 3, and 5 years, respectively. Most patients (90.6%) experienced rehospitalization during the follow-up period and the most common cause was LVAD-related infection. One-quarter of the patients (10 cases) required LVAD pump exchange, also most commonly due to LVAD-related infection. Comparative analysis with 213 patients who underwent LVAD implantation for bridge to transplant (BTT) revealed no significant change in the primary and secondary outcomes. Our results demonstrate favorable clinical outcomes for LVAD implantation for DT in this Japanese cohort.

**Supplementary Information:**

The online version contains supplementary material available at https://doi.org/10.1007/s10047-026-01581-8.

## Introduction

Left ventricular assist device (LVAD) implantation has drastically improved the survival and quality of life of patients with end-stage heart failure [1, 2]. Satisfactory outcomes have been reported for patients undergoing LVAD implantation for bridge to transplant (BTT), leading to its expanded indication as destination therapy (DT) for patient ineligible for heart transplantation in the United States and Europe. Favorable clinical results have been reported in DT cohorts, and the number and proportion of LVAD implantations for DT have increased over time [3, 4].

A new-generation LVAD, a fully magnetically levitated centrifugal-flow pump, has demonstrated superior event-free survival, particularly concerning thrombotic events, compared with earlier axial-flow pumps [5, 6]. However, despite the improvement safety and durability, most patients experience rehospitalization after implantation, often more than once. Furthermore, device-related infections and occasional heart failure exacerbations remain unresolved issues [3]. As a result, lifelong and optimal care for LVAD patients and the associated economic burden have become significant concerns in the DT era. LVAD implantation for DT was also accepted in Japan in 2021. Here, we report mid-term outcomes of LVAD implantation for DT at a single Japanese institution and this study will serve as a reference for future LVAD therapy in Japan.

## Materials and methods

### Study design and patient selection

Between April 2005 and March 2022, 253 patients underwent primary implantable left ventricular assist device (LVAD) implantation for end-stage heart failure at Osaka University Medical Hospital, Japan (Supplemental Fig. 1). Of these, 40 patients (15.8%) underwent LVAD implantation for destination therapy (DT). Among these 40 patients, 13 were definitive DT patients, whereas the remaining 27 patients, although technically categorized as bridge to transplantation (BTT), were considered de facto DT patients because of the donor allocation system in Japan. In the Japanese heart transplantation system during the study period, recipients younger than 60 years of age were prioritized for heart transplantation. In addition, renal or liver dysfunction, chronic lung disease, systemic inflammatory diseases such as systemic lupus erythematosus, and malignant tumors were regarded as relative or absolute contraindications for heart transplantation. Therefore, although these 27 patients were formally listed for heart transplantation, their likelihood of receiving a donor heart was considered extremely low because of their lower priority status. Since the Japanese DT criteria include elderly patients aged > 65 years and patients considered unsuitable candidates for heart transplantation, these 13 patients were categorized as definitive DT patients in the present study.

The postoperative observation period in this DT cohort was 36 months (6.5–54.4 months). The primary endpoint was on-device survival, and the secondary endpoints were LVAD-related complications during follow-up. Comparative analysis with 213 patients who underwent LVAD implantation for BTT at our institution was performed. Details of device selection, implantation techniques, and data collection are provided in the Supplemental Material.

### Statistical analysis

Continuous data are presented as median and interquartile values (the 1st quartile to the 3rd quartile). The Mann-Whitney U test was used for statistical comparisons. Longitudinal changes in the functional status were analyzed using linear mixed-effects models. Categorical variables presented as counts and percentages of patients, were compared using the χ^2^ test. When the expected frequency was less than 1 or 20% of the expected frequencies were less than or equal to 5, Fisher’s exact test was used. Cumulative survival curves and actuarial freedom from the event were computed using the Kaplan-Meier method, and compared using the log-rank test. Hazard ratios were calculated using Cox proportional hazards models. Statistical significance was set at P value < 0.05. All data were analyzed using the Statistical Analysis Systems software JMP Pro 16 (SAS Institute Inc., Cary, North Carolina, USA).

### Ethical approval

This study was approved by the Institutional Ethics Committee of Osaka University Medical Hospital (No. 16105; date February 11, 2016) and conducted in accordance with the 1964 Declaration of Helsinki and its later amendments. Written informed consent was obtained from all patients.

## Results

### Patient demographics

The patient details are presented in Table 1. The age at operation (62 [61–64] vs. 43 [32–52] years, *P*<0.0001) and HMRS (1.57 [1.03–2.03] vs. 0.92 [0.24–1.58], *P*<0.0001) were significantly higher in the DT group than in the BTT group. There were no significant differences in the etiology of cardiomyopathy, echocardiographic findings, cardiac function on right heart catheterization (RHC), or INTERMACS profile level between the two groups.

**Table 1:** Patients demographics

Age, years	DT (*n* = 40)	BTT (*n* = 213)	*P*-value
	62 (61–64)	43 (32–52)	< 0.0001
Female,** n**	13 (32.5%)	67 (31.5%)	0.90
Body surface area, m^2^	1.59 (1.43–1.70)	1.62 (1.50–1.73)	0.25
INTERMACS profile			0.59
Profile 1, n	2 (5.0%)	13 (6.1%)	
Profile 2, n	16 (40.0%)	72 (33.8%)	
Profile 3, n	14 (35.0%)	84 (39.4%)	
Profile 4, n	0 (0%)	10 (4.7%)	
Bridge to bridge, n	8 (20.0%)	34 (16.0%)	
Etiology of heart failure			0.21
Dilated cardiomyopathy, n	22 (55.0%)	118 (55.4%)	
Ischemic cardiomyopathy, n	10 (25.0%)	24 (11.3%)	
Hypertrophic cardiomyopathy, n	5 (12.5%)	31 (14.6%)	
Others, n	3 (7.5%)	40 (18.9%)	
Laboratory data			
Hemoglobin, g/dl	11.1 (9.8–12.6)	11.6 (10.2–13.2)	0.18
Creatinine, mg/dl	1.02 (0.77–1.45)	0.93 (0.73–1.16)	0.20
Total bilirubin, mg/dl	0.9 (0.5–1.4)	0.9 (0.6–1.4)	0.78
Albumin, g/dl	3.7 (3.1–4.0)	3.8 (3.3–4.2)	0.09
Brain natriuretic peptide, pg/ml	387.5 (220.8–661.0)	388.0 (166.0–692.4)	0.78
Echocardiography			
LVDd, mm	70 (58–78)	69 (60–78)	0.96
LVDs, mm	64 (54–74)	64 (54–73)	0.92
LVEF, %	18 (12–26)	20 (15–27)	0.10
Right heart catheterization			
Cardiac index, l/min/m^2^	1.96 (1.72–2.28)	2.09 (1.76–2.42)	0.19
Mean PAP, mmHg	31 (18–37)	27 (19–35)	0.13
PCWP, mmHg	21 (14–27)	19 (12–25)	0.36
Mean RAP, mmHg	6.5 (3.8–11.0)	7.0 (4.0–10.0)	0.71
HMRS	1.57 (1.03–2.03)	0.92 (0.24–1.58)	< 0.0001

*DT* destination therapy, *BTT* bridge to transplant, *LVDd* left ventricular diastolic diameter, *LVDs* left ventricular systolic diameter, *LVEF* left ventricular ejection fraction, *PAP* pulmonary artery pressure, *PCWP* pulmonary capillary wedge pressure, *RAP* right atrial pressure, *HMRS* Heart Mate II Risk Score

### Operative and perioperative results

Table 2 presents the operative and postoperative findings. The operative time was significantly longer in the DT group than in the BTT group (459 [351–550] vs. 373 [313–468] min, *P* = 0.007), although the cardiopulmonary bypass time was not significantly different. There was a significant difference in the devices used between the two groups: the use of EVAHEART and DuraHeart was lower in the DT group than in the BTT group (*P* = 0.042). There was no significant difference in concomitant valve procedures between the two groups. An additional right ventricular assist device (RVAD) was implanted in four cases (10.0%) in the DT group, which was not significant (*P* = 0.69). Three of these patients were successfully weaned from RVAD after seven, eleven, forty-one days of support, respectively; the fourth patient died due to LVAD pump infection after 99 days on LVAD support. The proportion of patients who underwent redo surgery was significantly higher in the DT group than in the BTT group (18 [45.0%] vs. 60 [28.2%], *P* = 0.034). The amounts of red cell concentrate, fresh frozen plasma and platelets transfusion during surgery were significantly higher in the DT group (14 [10–24] vs. 10 [6–18] units, *P* = 0.039, 15 [10–20] vs. 12 [8–18] units, *P* = 0.008 and 40 [20–56] vs. 20 [20–40] units, *P* = 0.006, respectively). The median length of intubation period (5.5 [2–14.5] vs. 2 [1–7] days, *P* = 0.009) and intensive care unit stay (12 [7–24] vs. 7 [4–15.5] days, *P* = 0.009) after surgery was significantly longer in the DT group than in the BTT group; however, there was no difference between the two groups in the length of hospital stay (*P* = 0.29). We experienced six of hospital death (15.0%) in the DT group, which was not significant compared with the BTT cohort (*P* = 0.33). Three of these patients died due to cerebral hemorrhage, two died due to sepsis derived by early pump pocket infection; the sixth died due to post-implantation, mediastinal bleeding-induced cardiac tamponade.

**Table 2:** Operative and post-operative data

Operation time, min	DT (*n* = 40)	BTT (*n* = 213)	*P*-value
	459 (351–550)	373 (313–468)	0.007
CPB time, min	161 (116–209)	147 (114–180)	0.16
Device			0.042
HeartMate3, n	5 (12.5%)	27 (12.6%)	
HVAD, n	3 (7.5%)	29 (13.6%)	
HeartMateII, n	19 (47.5%)	59 (27.7%)	
Jarvik2000, n	10 (25.0%)	29 (13.6%)	
EVAHEART, n	1 (2.5%)	33 (15.5%)	
DuraHeart, n	2 (5.0%)	36 (16.9%)	
Concomitant procedures			
Aortic valve procedure, n	8 (20.0%)	21 (9.9%)	0.06
Mitral valve procedure, n	5 (12.5%)	16 (7.5%)	0.29
Tricuspid valve procedure, n	10 (25.0%)	66 (31.0%)	0.45
Others, n	7 (17.5%)	20 (9.4%)	0.13
RVAD implantation, n	4 (10.0%)	26 (12.2%)	0.69
Redo surgery, n	18 (45.0%)	60 (28.2%)	0.034
Transfusion			
RCC, Units	14 (10–24)	10 (6–18)	0.039
FFP, Units	15 (10–20)	12 (8–18)	0.008
Platelets, Units	40 (20–56)	20 (20–40)	0.006
Intubation period, days	5.5 (2–14.5)	2 (1–7)	0.009
ICU stay, days	11 (7–24)	7 (4–15.5)	0.009
Hospital stay, days	110 (77–162)	92 (67–145)	0.29
NO usage, n	33 (82.5%)	166 (77.9%)	0.52
Re-exploration for bleeding, n	14 (35.0%)	54 (25.4%)	0.21
Temporary RRT, n	8 (20.0%)	28 (13.2%)	0.25
Tracheostomy, n	18 (45.0%)	37 (17.4%)	0.0001
Hospital death, n	6 (15.0%)	21 (9.9%)	0.33

*DT* destination therapy, *BTT* bridge to transplant, *CPB* cardiopulmonary bypass, *RVAD* right ventricular assist device, *RCC* red cell concentrate, *FFP* fresh frozen plasma, *ICU* intensive care unit, *NO* nitric oxide, *RRT* renal replacement therapy

### Clinical outcomes

**Fig. 1:**
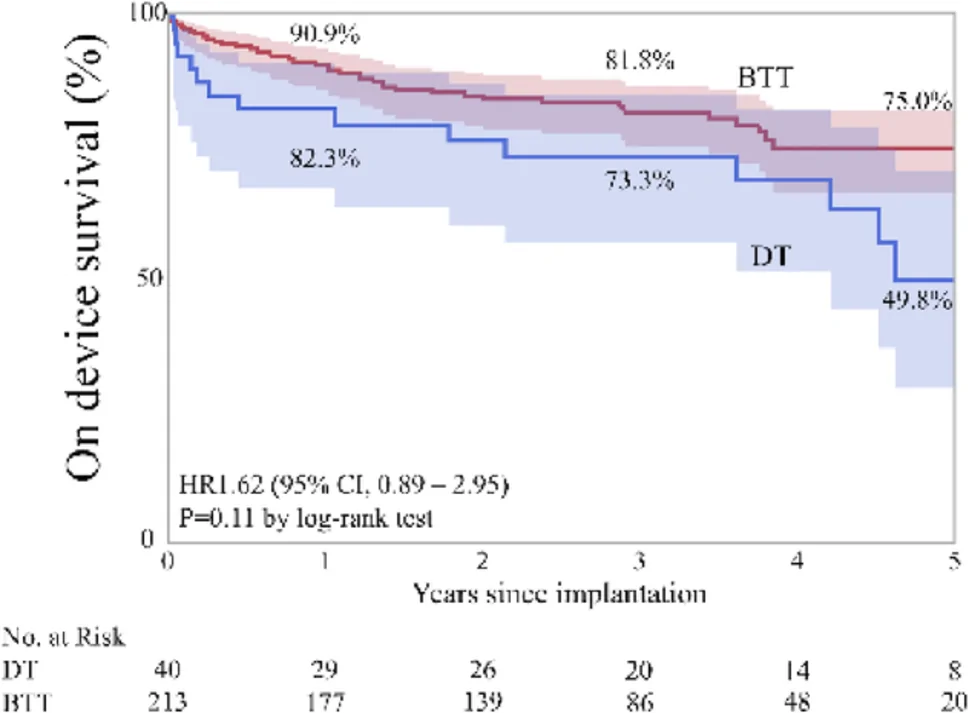
Kaplan–Meier estimates of survival after left ventricular assist device implantation. DT, destination therapy; BTT, bridge to transplant

**Fig. 2:**
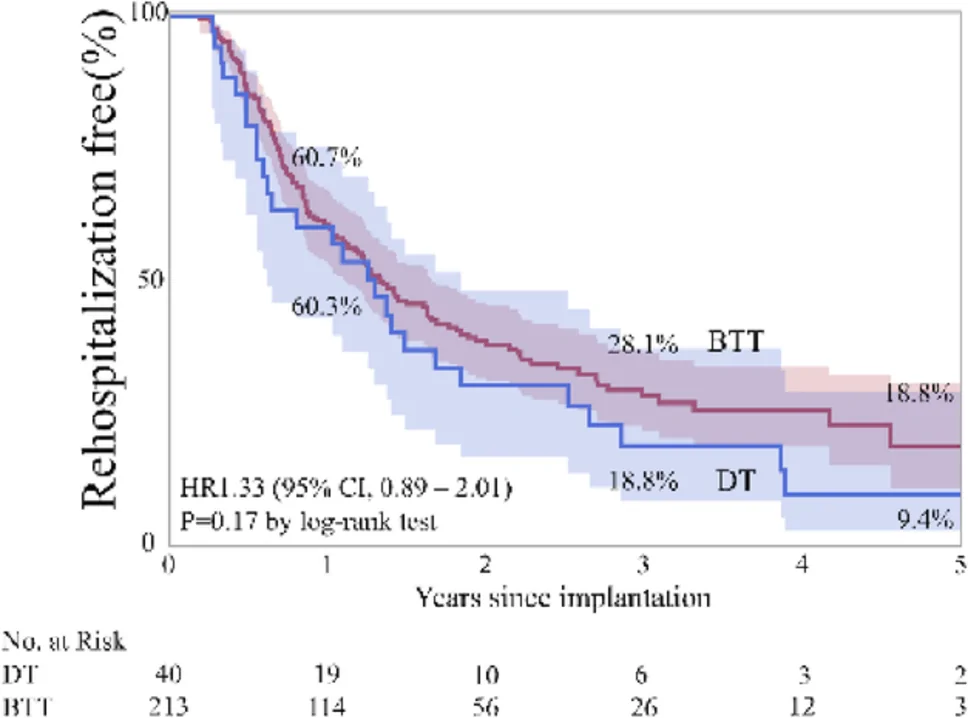
Kaplan–Meier estimates of freedom from rehospitalization after left ventricular assist device implantation. DT, destination therapy; BTT, bridge to transplant

On- device survival is demonstrated and compared in Fig. 1. The survival rates of the DT group during the observation period were 82.3%, 73.3% and 49.8% at 1-, 3-, and 5-years follow-up; there was no significant change between the DT and BTT groups. The survival freedom from rehospitalization after the first discharge during follow-up is shown in Fig. 2, with rates of 60.3%, 18.8%, and 9.4% at 1-, 3-, and 5-year follow-up. There was no significant change the DT and BTT groups. Almost half of the patients experienced rehospitalization due to infection. The details of the post-implantation LVAD-related complications are shown in Supplemental Fig. 2. No significant difference was observed in all the LVAD-related complications between the DT and BTT groups. A total of 10 patients (25%) in the DT group underwent LVAD pump exchange during follow-up period. The details of the patients who underwent LVAD pump exchange and operative results are shown in Supplemental Tables 1 and 2. The median duration until pump exchange was 788 days (141–1474 days) after the first LVAD implantation. All procedures were successfully performed; however, we experienced one (10%) hospital death case due to cerebral hemorrhage. Among the patients initially classified into the DT group, three patients were subsequently converted to BTT during follow-up. All three patients were older than 60 years and eventually underwent heart transplantation after accepting marginal donors because of LVAD-related complications, including pump thrombosis and driveline infection.

## Discussion

The primary findings of this study were as follows: (1) There was no significant difference in the on-device survival and LVAD-related complication-free survival between the DT and BTT groups, although the operative risk was relatively high in the DT group. (2) Most patients in the DT cohort experienced rehospitalization after the first discharge, within a median duration of 269 (135–660) days, and the most significant cause was LVAD-related infection. (3) One-quarter of the patients in the DT group required additional LVAD exchange due to device-related complications during the observational period.

We presented a single Japanese institutional experience on LVAD implantation for DT in a series of 40 patients. The short-term survival rate was satisfactory, as the on-device survival rate was 82.3% at 1 year and 73.3% at 3 years in our institute experience. Although the DT group had substantially higher baseline risk, including advanced age and higher HMRS, mid-term survival was not significantly different from that in the BTT group. This finding may suggest that although DT patients are clinically more fragile, acceptable long-term outcomes can be achieved if the acute postoperative phase is appropriately managed. Indeed, postoperative tracheostomy was significantly more frequent in the DT group, and re-exploration for bleeding as well as temporary renal replacement therapy also tended to occur more frequently, although these differences did not reach statistical significance. These findings indicate that DT patients undoubtedly represent a high-risk population requiring intensive perioperative management. However, timely postoperative interventions, including aggressive respiratory and organ support when necessary, may contribute to favorable long-term outcomes even in high-risk DT patients. Previous reports demonstrated poorer patient backgrounds, especially regarding organ dysfunction, with a 2-year survival rate of approximately 58% [2]. However, with the expansion of DT indications, the impact of unfavorable patient characteristics other than age on clinical outcomes appears to have diminished [7, 8]. A similar trend was observed in our study. In addition, optimization of perioperative management and improvements in LVAD technology may also have contributed to the favorable outcomes in the DT population.

In our study, the early death rate in the DT group seemed to be higher compared with the BTT group. This may have primarily been due to the higher proportion (25%) of high-risk patients, including INTERMACS profile 1 and bridge to bridge patients, in this DT cohort. Six patients (15%) died during the index hospitalization in our study ; two of them (33.3%) were in the bridge to bridge group. Since the critically-ill patients have poorer prognosis, the indication for DT in these patients have decreased with an interest in earlier LVAD implantation in the future [9]. Recent reports from the contemporary HeartMate3 (Abbott Laboratories, Chicago, Illinois, USA) era have proposed the concept of an “LVAD-first” or sequential strategy, in which durable LVAD support followed by delayed heart transplantation may maximize lifetime survival in selected patients with advanced heart failure [10, 11]. In our study, despite the relatively limited use of HeartMate3, the BTT cohort demonstrated a 2-year survival rate of 85.0%, which was comparable to previously reported outcomes after heart transplantation and HeartMate3 support, particularly with respect to survival during the transplant waiting period from United Network for Organ Sharing database [11]. These findings may suggest that, regardless of DT or BTT indication, the current era favors considering LVAD implantation at an earlier stage rather than waiting until patients become critically ill — namely, at a stage where favorable outcomes can still be expected, such as INTERMACS profiles 3–4 and selected profile 5–6 patients. Since heart transplantation itself is not necessarily a lifelong solution because of complications such as renal dysfunction and infection, broader consideration of durable LVAD strategies may be reasonable, especially in Japan where donor shortage remains a major issue. Nevertheless, frequent rehospitalizations and persistent LVAD-related complications continue to represent important unresolved challenges.

During the observational period, almost all patients in the DT experienced rehospitalization. This is a big concern because rehospitalization could be a mental and financial burden on patients and their families [12]. The most common causes for rehospitalization were LVAD-related infection. LVAD-related infection is a serious problem in the DT population because heart transplantation is not feasible, sometimes requiring invasive pump exchange. To slow the progression of infection and reduce pump exchange, early diagnosis and intervention are critical.

In patients with LVAD, pump exchange is sometimes required for several reasons, such as pump thrombosis or LVAD-related infection or device failure. Pump exchange is usually highly invasive, especially in elderly patients. Several reports demonstrated almost the same survival rate in patients who underwent LVAD pump exchange compared with primary LVAD patients [13, 14]. The reported rate of hospital death is 10% and is also not significant. However, a cause for pump exchange is important and patients who underwent pump exchange for LVAD-related infection appeared to have poorer prognosis and time to exchange after diagnosis of infection influenced to survival [14]. In our cohort, we experienced two death cases out (one was hospital death) of ten pump exchange cases, and all of two cases underwent pump exchange due to LVAD-related infection. In contrast, other patients who underwent pump exchange demonstrated good survival. Therefore, the physicians should consider pump exchange indications after careful consideration of the patients’ conditions.

## Limitations

This study was a descriptive retrospective analysis and was not hypothesis driven. In addition, there is a gap in the time context and patients’ backgrounds between de facto and definitive DT patients. Regarding whole cohort, Because the era of LVAD implantation and the distribution of implanted devices differed between the DT and BTT cohorts, the potential impact of these factors on clinical outcomes could not be completely excluded.

## Conclusions

Our study demonstrated satisfactory mid-term outcomes of LVAD implantation as DT in a Japanese cohort. There was no significant difference in survival and incidence of LVAD-related complications between the DT and BTT groups. Regarding the high rehospitalization rate and number of pump exchange cases, controlling LVAD-related infection is a future task.

## Supplementary Information

Below is the link to the electronic supplementary material.

Supplementary Material 1.

Supplementary Material 2.

Supplementary Material 3.

Supplementary Material 4.

Supplementary Material 5.

Supplementary Material 6.

Supplementary Material 7.

Supplementary Material 8.

Supplementary Material 9.

Supplementary Material 10.

## Data Availability

The deidentified participant data will not be shared.
