# Supplementary material for: Clinical differences between destination therapy and bridge-to-transplant left ventricular assist device implantation: a 17-year single-center experience in Japan
Source: J Artif Organs. 2026 Aug 21;29(3):58. doi: 10.1007/s10047-026-01581-8 (PMC13498637; doi:10.1007/s10047-026-01581-8)
Supplement: Supplementary file 1 [file 10047_2026_1581_MOESM1_ESM.docx]

**Supplemental Materials and methods**

**Device selection, implantation techniques, and postoperative management**

The device was selected according to the surgeon’s preference. The following devices were used: HeartMate3 (Abbott Laboratories, Chicago, Illinois, USA), HVAD (Medtronic, Dublin, Ireland), EVAHEART (Sunmedical Technology Research Corporation, Nagano, Japan), DuraHeart (Terumo Corporation, Tokyo, Japan), Jarvik2000 (Jarvik Heart, New York, New York, USA), and HeartMateII (Abbott Laboratories, Chicago, Illinois, USA). EVAHEART included the modified EVAHEART2 as well. LVAD implantation procedures were usually performed via a traditional median sternotomy. Bilateral mini-thoracotomy was performed in a limited number of patients for HeartMate3 and HVAD, following a previously reported approach [1]. The LVAD inflow cuff or pump was attached to the ventricular apex, generally under the beating heart. Cardiac arrest was induced by ante- or retro-grade cardioplegia after direct aortic cross-clamping in patients who underwent concomitant surgery, such as aortic or mitral valve procedures. Aortic valve procedures were performed with aortic valve replacement or aortic valve closure with the patch closure technique or park stitch technique according to the surgeon’s preference. Post-auricular maneuver, as previously described, was used for Jrvik2000 implantation in patients undergoing DT [2]. After operation, recommended antithrombotic treatment was initiated including daily aspirin at a dosage of 100mg and warfarin to a target range for an international normalized ratio of 2.0 to 3.0 in all cases by each device’s characteristics. Monthly medical checkups, including interviews of daily life, blood sampling, and wound (driveline exit site and incision sites) care, were usually performed after discharge, unless serious complications occurred.

**Data collection**

The primary outcome variables were the in-hospital and overall survival rates. Preoperative variables that may have affected the primary outcome were identified, including baseline demographics, laboratory values, and circulatory hemodynamics. Intraoperative variables, such as cardiopulmonary bypass time, amount of blood products used, and concomitant cardiac procedures were also collected. Early post-implantation data included complications that occurred between operation and hospital discharge. Major adverse events requiring rehospitalization during LAVD support were also identified. These included major cerebral events, driveline infections, LVAD pump pocket infection, pump thrombosis, gastrointestinal bleeding, LVAD-related arrhythmia, pump exchange, and heart failure. In the present study, cerebrovascular accident and LVAD-related infections including pump pocket infection, were defined as previously reported [3]. LVAD pump thrombosis was defined as highly suspicion based on clinical data, such as hemolysis, sudden elevation of serum lactate dehydrogenase level, and device malfunction, which required pump exchange or strengthened anticoagulant therapy. LVAD-related arrhythmia was defined as ventricular fibrillation or tachycardia first detected after LVAD implantation on electrocardiography, that required cardioversion, additional intravenous or oral antiarrhythmic agents, or catheter ablation. Heart failure was defined as the exacerbation of left or right heart failure which required rehospitalization, or additional catecholamine or diuretic use. The Heart Mate II Risk Score (HMRS) was calculated as follows: HMRS = 0.0274 × age - 0.723 × serum albumin (mg/dL) + 0.74 × serum creatinine (mg/dL) + 1.136 × INR.

**References**

[1] Popov AF, Hosseini MT, Zych B, Simon AR, Bahrami T. HeartWare left ventricular assist device implantation through bilateral anterior thoracotomy. Ann Thorac Surg. 2012; 93(2): 674-676.

[2] Carrozzini M, Bejko J, Gregori D, Gerosa G, Bottio T. How to implant the Jarvik 2000 post-auricular driveline: evolution to a novel technique. J Artif Organs. 2019; 22(3): 188-193.

[3] Yoshioka D, Toda K, Ono M, Nakatani T, Shiose A, Matsui Y, et al. Clinical Results, Adverse Events, and Change in End-Organ Function in Elderly Patients with HeartMateII Left Ventricular Assist Device – Japanese Multicenter Study. Circ J. 2018; 82(2): 409-418.

**Table 1: Demographics of patients with pump exchange**

| **No.** | **Etiology** | **Sex** | **Age at 1^st^ LVAD (years)** | **1^st^ LVAD support duration (days)** | **Reason for pump exchange** | **1^st^ device** |
| --- | --- | --- | --- | --- | --- | --- |
| **1** | dHCM | Female | 64 | 36 | Pump thrombosis | HMII → DuraHeart |
| **2** | DCM | Male | 60 | 2194 | Pump infection | HMII → HM3 |
| **3** | DCM | Female | 63 | 1587 | Device failure | HMII → HM3 |
| **4** | dHCM | Male | 59 | 935 | Driveline infection | Jarvik2000 → Jarvik2000 |
| **5** | dHCM | Female | 65 | 241 | Device failure | HMII → HMII |
| **6** | DCM | Male | 61 | 1169 | Driveline infection | HMII → HM3 |
| **7** | ICM | Male | 61 | 1429 | Driveline infection | HMII → HM3 |
| **8** | DCM | Male | 69 | 589 | Driveline infection | HVAD → HVAD |
| **9** | ICM | Female | 64 | 46 | Pump infection | HVAD → HVAD |
| **10** | ICM | Male | 65 | 533 | Driveline infection | HVAD → HVAD |

LVAD, left ventricular assist device; dHCM, dilated-phase hypertrophic cardiomyopathy; DCM, dilated cardiomyopathy; ICM, ischemic cardiomyopathy; HMII, HeartMateII; HM3, HeartMate3.

**Table 2: Perioperative details of pump exchange patients**

| **No.** | **Approach** | **Op time**  **(min)** | **Concomitant surgeries** | **Transfusion**  **RBC/FFP/Plt (units)** | **In hospital complications** | **ICU stay (days)** | **Hospital stay (days)** | **Outcome** |
| --- | --- | --- | --- | --- | --- | --- | --- | --- |
| **1** | Full sternotomy | 649 | None | 30/28/60 | Cerebral hemorrhage  SSI  Re-exploration  Tracheostomy | 23 | 297 | Heart transplant |
| **2** | Full sternotomy | 533 | Temporary RVAD implantation | 34/32/60 | Cerebral hemorrhage  Pump infection  Re-exploration  Tracheostomy | 54 | 143 | Hospital death  (cerebral bleeding) |
| **3** | Full sternotomy | 343 | None | 24/14/40 | None | 7 | 61 | Outpatient |
| **4** | Thoracotomy | 566 | None | 10/16/40 | GI bleeding | 10 | 92 | Death (604 days after pump exchange)  (cerebral bleeding) |
| **5** | Thoracotomy | 224 | None | 16/10/20 | None | 6 | 24 | Outpatient |
| **6** | Full sternotomy | 402 | None | 16/12/20 | GI bleeding | 7 | 50 | Outpatient |
| **7** | Full sternotomy | 484 | AV closure | 10/26/40 | Mediastinitis  Re-exploration | 48 | In the hospital | In the hospital |
| **8** | Full sternotomy | 668 | Omental flap transposition | 44/42/60 | Tracheostomy | 29 | 99 | Outpatient |
| **9** | Full sternotomy | 587 | Omental flap transposition | 30/28/60 | Heart failure  Re-exploration  Tracheostomy | 44 | 189 | Outpatient |
| **10** | Full sternotomy | 686 | Omental flap transposition | 44/32/40 | None | 6 | 39 | Outpatient |

Op, operation; RVAD, right ventricular assist device; AV, aortic valve; RBC, red blood concentrate; FFP, fresh frozen plasma; Plt, platelets; SSI, surgical site infection; GI bleeding, gastrointestinal bleeding; ICU, intensive care unit.

**Supplemental Figure Legends**

**Figure 1.**

Temporal distribution of LVAD implantation in the DT and BTT cohorts according to implantation year.

DT, destination therapy; BTT, bridge to transplant.

**Figure 2.**

Kaplan-Meier model of freedom from left ventricular assist device related (LVAD) complications.

(A) Driveline exit-site infection free survival. (B) Pump infection free survival. (C) Cerebrovascular accident (CVA) free survival. (D) LVAD pump thrombosis free survival. (E) Gastrointestinal bleeding free survival. (F) LVAD related arrhythmia free survival. (G) Exacerbation of heart failure (HF) free survival. (H) LVAD pump exchange free survival.

DT, destination therapy; BTT, bridge to transplant.
